# Bradycardia Leading to Asystole Following Dexmedetomidine Infusion during Cataract Surgery: Dexmedetomidine-Induced Asystole for Cataract Surgery

**DOI:** 10.1155/2018/2896032

**Published:** 2018-12-05

**Authors:** Amaniti Aikaterini, Dalakakis Ioannis, Gkinas Dimitrios, Sapalidis Konstantinos, Grosomanidis Vasilios, Papazisis George

**Affiliations:** ^1^AHEPA University Hospital, Aristotle University of Thessaloniki, Thessaloniki, Greece; ^2^Laboratory of Pharmacology, Aristotle University of Thessaloniki, Thessaloniki, Greece

## Abstract

Dexmedetomidine is an elective alpha-2 adrenergic agonist, being used in anesthesia practice. It offers sedative, anxiolytic, analgesic, muscle relaxant, and sympathetic lytic properties. While respiratory effects are considered minor, there are important cardiovascular side effects with bradycardia and hypotension being the most common. This article presents a case of a patient, with significant comorbidities and polypharmacy, who was scheduled for cataract surgery under dexmedetomidine sedation. During the administration, severe hemodynamic deterioration was observed with bradycardia and hypotension leading to asystole. Cardiac arrest was successfully managed in accordance with the guidelines of the European Resuscitation Council. Despite the promising role of dexmedetomidine in anesthesia practice, the associated adverse effects cannot be ignored. For this reason it is obligatory to have the minimum mandatory monitoring in accordance with the safety and quality guidelines.

## 1. Introduction

Dexmedetomidine is unique for its sedative and anxiolytic properties in the operating room or intensive care unit, due to its minimal respiratory effects [1]. Dexmedetomidine-induced cardiovascular effects are usually characterized by lowered heart rate and blood pressure [2, 3]. While cases of bradycardia or asystole under dexmedetomidine have been documented [4], asystole has been perpetuated by either the coexistence of factors like spinal anesthesia [4] or increased doses [5]. Moreover, little is known about the interaction of dexmedetomidine with other medications. We describe a case of bradycardia and asystole, following dexmedetomidine infusion, in a patient undergoing cataract surgery.

## 2. Case Report

A male patient, 54 years old, 170 cm, 80 kg, American Society of Anesthesiologist Physical Status (ASA-PS) graded 3, was planned for elective cataract surgery. Previous medical history of the patient revealed severe psychotic disorder and drug-resistant epilepsy, diagnosed by the age of 6. He also suffered from congenital bilateral nerve palsy and stinging, type II diabetes mellitus, and dyslipidemia. Smoking, alcohol abuse, or allergies were not reported. Patient's activity, estimated by metabolic equivalents (METS), could not be assessed. His current medication included Levetiracetam mg 1500 mg twice daily, Oxcarbazepine 400 mg twice daily, Clobazam 10 mg once daily, Ebastine 20 mg every other day, Pregabalin mg 150 mg three times daily, Risperidone 2 mg twice daily, Metformin 500 mg twice daily, Ezetimibe 10 mg once daily, Eicosapentaenoic acid 1000 mg once daily, and Omeprazole 20 mg once daily.

Due to poor communication and cooperation, the ophthalmologist in charge decided to operate the patient under general anesthesia. During preoperative screening, clinical examination did not reveal pathological findings, ECG was reported without specific lesions, and chest X-ray was normal. Preoperative airway evaluation revealed prognostic factors of difficult airway: Mallampati classification grade III, thyromental distance (TMD) <6cm, median teeth distance <3cm, and moderate cervical spine mobility. Due to anticipated difficult intubation and based on the poor physical status, a sedation technique with dexmedetomidine was decided instead of general anesthesia.

After standard monitoring and intravenous line establishment, the patient was prehydrated with 300 ml of Ringer's Lactate, followed by a single bolus of 50 mcg of Fentanyl as an adjuvant analgesic.

Infusion of dexmedetomidine started at a loading dose of 1 mcg/kg for 10 minutes, followed by a maintenance dose of 0.4 mcg/kg/h. However, after 17 minutes of commencement of dexmedetomidine infusion and before surgery, the patient suddenly suffered bradycardia with hemodynamic collapse. Blood pressure was 75/45 mmHg and heart rate 40 bpm. Immediately 0.5 mg of atropine was administered and infusion of dexmedetomidine stopped at the same time. Seconds after, cardiac arrest with asystole occurred. Advanced life support and cardiopulmonary resuscitation was implemented according to the European Resuscitation Council algorithm. Return of spontaneous circulation (ROSC) occurred at about 4 minutes after 2 cycles of cardiopulmonary resuscitation. The operation was postponed. When the patient gained satisfactory neurological status of consciousness, he was transferred to the Coronary Unit for further intensive care. Few hours later he was transferred to the ward in good overall condition.

## 3. Discussion

Bradycardia and asystole after dexmedetomidine infusion is a well-described phenomenon [4]. Some of them have been attributed to the administered doses or surgical stimulation, while other authors consider cardiac morbidity as a cofactor.

In our case, dexmedetomidine was administered at a dose of 1 mcg/kg for 10 minutes followed by continuous infusion at a rate of 0.4 *μ*g/kg/h. Based on a model of simulated concentration, a concentration of 0.62ng/ml was estimated for our patient [4]. Depression of cardiac function would have been induced when the plasma concentration of dexmedetomidine exceeded 1.2 ng/ml [5] although plasma concentration of dexmedetomidine was not measured in this case. Takada et al. concluded that risk of cardiac arrest is not necessarily associated with the blood concentration of dexmedetomidine [4].

It is worthwhile to note that our concerns were about polypharmacy and the potential interactions between the patient's current medication and dexmedetomidine. There is no data in the literature regarding the coadministration of dexmedetomidine with many other drugs. However, the combination of dexmedetomidine with benzodiazepines has been used for the treatment of alcohol withdrawal syndrome. Double blind randomized trials demonstrated a statistically better outcome in the group receiving dexmedetomidine and benzodiazepines compared to the benzodiazepine monotherapy group. Nevertheless, bradycardia was reported as a common event with dexmedetomidine [6].

Another key point is the possible interaction between dexmedetomidine and pregabalin. While there are no reports regarding their coadministration, pregabalin has been found to lower heart rate and attenuate sympathetic outflow during laryngoscopy [7].

The introduction of dexmedetomidine offers the anesthetist many advantages without causing respiratory depression. But it does not lack other side effects with bradycardia being the prominent one. For this reason, it is mandatory to have a standard monitoring as well as the alertness to manage critical incidents.
