# Corrigendum to “Bradycardia Leading to Asystole Following Dexmedetomidine Infusion during Cataract Surgery: Dexmedetomidine-Induced Asystole for Cataract Surgery”

**DOI:** 10.1155/2019/7254218

**Published:** 2019-10-29

**Authors:** Aikaterini Amaniti, Ioannis Dalakakis, Dimitrios Gkinas, Konstantinos Sapalidis, Vasilios Grosomanidis, Georgios Papazisis

**Affiliations:** ^1^AHEPA University Hospital, Aristotle University of Thessaloniki, Thessaloniki, Greece; ^2^Laboratory of Pharmacology, Aristotle University of Thessaloniki, Thessaloniki, Greece

In the article titled “Bradycardia Leading to Asystole Following Dexmedetomidine Infusion during Cataract Surgery: Dexmedetomidine-Induced Asystole for Cataract Surgery” [1], the names of some of the authors were given incorrectly as “Amaniti Aikaterini, Dalakakis Ioannis, Sapalidis Konstantinos, Grosomanidis Vasilios, and Papazisis George”. The authors' names should have been written as “Aikaterini Amaniti, Ioannis Dalakakis, Konstantinos Sapalidis, Vasilios Grosomanidis, and Georgios Papazisis”. The revised authors' list is shown above.
